# “Is multipoint pacing superior to optimized single-point pacing?”—Authors’ reply

**DOI:** 10.1111/jce.15276

**Published:** 2021-10-26

**Authors:** Vishal S. Mehta, Mark K. Elliott, Baldeep S. Sidhu, Justin Gould, Bradley Porter, Steven Niederer, Christopher A. Rinaldi

**Affiliations:** 1 Cardiology Department, Guy’s and St Thomas’ NHS Foundation Trust, London, UK; 2 School of Biomedical Engineering and Imaging Sciences, King’s College London, London, UK

We read with interest the letter from colleagues San Antonio et al.^1^ We acknowledge that the scope of the meta-analysis was limited to multipoint pacing (MPP) without the potential benefit of fusion optimized intervals (FOI) methods being implemented. The a priori inclusion criteria and scope of our review did not encompass this.

San Antonio et al. highlight that utilizing FOI by altering the atrioventricular (AV) and interventricular (VV) delays to achieve fusion of the paced and intrinsic conduction may improve QRS duration (QRSd). This may in turn lead to improved coordinated contraction of the ventricles. The two studies highlighted utilize FOI with and without MPP and compare FOI directly to MPP only—demonstrating no clear benefit of using MPP in these circumstances.^1,2^

While FOI methods are one technique of potentially optimizing CRT delivery, there are limitations. First, the method involves optimization of intervals at rest which does not consider physiological variations based on individual patient activity and characteristics, the risk of pseudo-fusion related to changes in intrinsic AV times, and whether those with prolonged AV delays are appropriate for the method. Second, as illustrated in a contemporaneous review,^3^ FOI has only been tested for left ventricular reverse remodeling in a single-center RCT involving 180 patients.^4^ The remaining studies utilize a variety of different study designs and primarily use an acute endpoint such as QRSd reduction or acute hemodynamic response to evaluate FOI efficacy. This makes evaluating the clinical efficacy of FOI challenging. Third, in optimizing AV delay to achieve synchronous activation, it is likely that optimal filling will not be achieved.^5^ This may have detrimental effects on contractility. It is not clear how to set AV delay to balance electrical synchrony and time filling to optimize CRT response.

We feel our review systematically and objectively evaluated the different studies available in the field of MPP. San Antonio et al. appropriately identifies alternative methods of CRT optimization, however, in our opinion, FOI requires further data from randomized trials evaluating longer-term clinical outcomes before it can be compared to alternative methods of RT delivery, including MPP. MPP continues to promise further large-scale trial results soon.^6^ FOI methods require similar assessment.
